# Postoperative Infection After Esophageal Injury in Anterior Cervical Spine Surgery: A Comprehensive Review of Diagnosis, Management, and Outcomes

**DOI:** 10.3390/jcm14093244

**Published:** 2025-05-07

**Authors:** Chae-Gwan Kong, Jong-Beom Park

**Affiliations:** Department of Orthopaedic Surgery, Uijeongbu St. Mary’s Hospital, College of Medicine, The Catholic University of Korea, Uijeongbu 11765, Republic of Korea; gongjae@catholic.ac.kr

**Keywords:** postoperative complications, esophageal injury, cervical spine infection, surgical management, infection control, rehabilitation

## Abstract

Postoperative infection following anterior cervical spine surgery, particularly when complicated by esophageal injury, is a rare but serious condition associated with significant morbidity and mortality. This review elucidates the complex interplay between postoperative infection and esophageal injury. We systematically analyzed studies from 2000 to 2025 using PubMed, Scopus, and Web of Science, focusing on infection, esophageal injury, surgical outcomes, and management strategies, with emphasis on recent advances in diagnostics, surgical techniques, and postoperative care. Our findings highlight the multifactorial nature of these complications and the critical role of early recognition, accurate diagnosis, and timely management. Imaging modalities such as CT, MRI, and contrast esophagography, along with flexible esophagoscopy, are indispensable in assessing injury and infection extent. Effective management requires a multidisciplinary approach integrating broad-spectrum antibiotics, surgical debridement, vascularized flap reinforcement, negative pressure wound therapy, and antibiotic-loaded cement beads. Meticulous postoperative care with prolonged antibiotics, nutritional support, and imaging follow-up is vital for optimizing outcomes. Innovative approaches, including vascularized muscle flaps and hyperbaric oxygen therapy, show promise in enhancing healing and reducing infections. Our review underscores the need for future meta-analyses to strengthen evidence and refine protocols. As surgical techniques evolve, so too must our diagnostic, surgical, and postoperative strategies to minimize complications and improve patient outcomes.

## 1. Introduction

**Figure 1:**
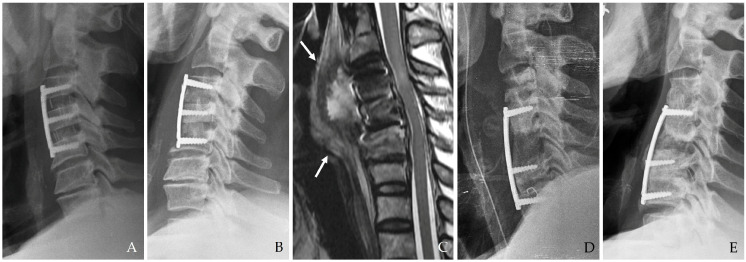
The patient underwent C3-4-5 anterior cervical discectomy and fusion (**A**). At postoperative 2 months, postoperative infection occurred with retropharyngeal abscess (white arrows) and epidural abscess (**B**,**C**). Therefore, the patient underwent evacuation of retropharyngeal and epidural abscesses, irrigation/debridement, and revisional C5-6-7 corpectomy and fusion (**D**) and achieved a cure for the infection (**E**).

Anterior cervical spine surgery is a critical intervention for a variety of conditions ranging from degenerative disc diseases and traumatic injuries to neoplastic disorders. Although significant improvements have been achieved in surgical techniques, perioperative infection control, and postoperative care, the risk of postoperative infection remains a serious concern [1,2,3]. While the overall incidence of infection in cervical spine surgery is relatively low compared to lumbar procedures due to superior local vascularity and less extensive soft tissue dissection, the anatomical proximity of the cervical spine to vital neurovascular structures and the esophagus creates a high-risk environment for severe complications (Figure 1) [1,2,4,5].

The presence of concomitant esophageal injury significantly increases the risk of postoperative infection, as the breach in the natural barrier permits the entry of oral and gastrointestinal flora into the surgical field [6,7]. Such contamination can lead to a range of deep infections, including mediastinitis, abscess formation, osteomyelitis, epidural abscess, sepsis, and even death [6,7]. The clinical manifestations of these infections are highly variable, ranging from localized symptoms such as erythema, wound drainage, and dysphagia to severe systemic disturbances, including fever, neurological deficits, and respiratory distress [7,8]. Without timely diagnosis and intervention, these infections may progress rapidly, resulting in catastrophic outcomes [8].

Epidemiological studies indicate that the incidence of postoperative infection following anterior cervical spine surgery ranges from 0.1% to 1.6% [1,2,4]. However, the rate increases significantly when esophageal injury is involved, with reports indicating incidences as high as 6.5% in cases with esophageal perforation [6,7]. Risk factors for postoperative infection include prolonged operative time, multilevel procedures, revision surgeries, immunosuppression, and preexisting comorbidities such as diabetes and obesity [2,5].

Due to the nature of this topic, conducting a systematic review or meta-analysis presents significant challenges [1,4]. The available studies on postoperative infection following anterior cervical spine surgery exhibit considerable heterogeneity in terms of study design, patient populations, surgical techniques, and outcome measures [1,4]. Furthermore, many reports are descriptive or observational in nature, consisting of case series, cohort studies, and expert opinion articles rather than randomized controlled trials (RCTs) [1,4]. Consequently, our review adopts a narrative approach aimed at providing a comprehensive overview of the existing literature, synthesizing expert opinions, and incorporating instructive cases to enhance clinical understanding [1,4]. While a meta-analysis could potentially offer additional insights, the current evidence base lacks the uniformity required for rigorous quantitative analysis. We acknowledge the value of conducting such a study in the future, particularly as more high-quality, prospective studies become available [1,4]. As such, we have highlighted this consideration within the discussion section as a promising area for future research.

The purpose of this comprehensive review is to elucidate the complex interplay between postoperative infection and esophageal injury in anterior cervical spine surgery. We aim to provide spine surgeons with practical insights into early diagnosis, effective treatment strategies, and long-term follow-up care to improve clinical outcomes and minimize complications.

## 2. Methodology

We systematically reviewed studies published from 2000 to 2025 using PubMed, Scopus, and Web of Science databases. Search terms included ‘postoperative infection’, ‘anterior cervical spine surgery’, ‘esophageal injury’, and ‘treatment strategy’. Inclusion criteria focused on relevance to the topic, study design, and quality. Articles addressing postoperative infections associated with anterior cervical spine surgery, particularly those involving esophageal injury, were selected for detailed analysis. Exclusion criteria included studies that did not specifically address postoperative infection management in the context of anterior cervical spine surgery or lacked adequate methodological rigor.

Due to the heterogeneity in available studies, we adopted a narrative approach to provide a comprehensive overview of the existing literature. Observational studies, cohort studies, case series, and expert opinions were included to enhance clinical understanding. While a meta-analysis could potentially offer additional insights, the current evidence base lacks uniformity for rigorous quantitative analysis. Future research should focus on conducting high-quality prospective studies and meta-analyses to establish evidence-based protocols.

## 3. Clinical Presentation

The clinical presentation of postoperative infection following anterior cervical spine surgery complicated by esophageal injury is multifaceted and may vary widely based on the timing of onset, injury severity, extent of infection spread, and presence of comorbid conditions. Rapidly recognizing these early and late symptoms is essential because delayed diagnosis significantly increases the risk of irreversible damage, life-threatening complications, and prolonged hospital stays [9,10,11,12,13,14,15,16]. The clinical manifestations can range from mild, localized symptoms to severe, systemic conditions depending on the extent and location of the infection.

### 3.1. Early-Onset Symptoms (Within 24–72 h Postoperatively)

In the immediate postoperative period, rapid onset of symptoms is typical due to direct surgical field contamination and breaches in the natural protective barriers [9]. Key early clinical features include the following.

#### 3.1.1. Severe Dysphagia and Odynophagia

Patients often report intense pain and difficulty swallowing. This may rapidly escalate over the first 24 to 72 h, reflecting the acute inflammatory response and possible leakage of esophageal contents into surrounding tissues. Dysphagia is often progressive, worsening with each attempt to swallow, particularly when involving solid foods [9,10].

#### 3.1.2. Local Inflammatory Signs

Noticeable swelling, redness, tenderness, and warmth around the surgical incision are typical findings. These signs indicate localized soft tissue inflammation and an underlying infectious process. Erythema and induration may extend beyond the immediate surgical site, signifying the spread of the infection to adjacent structures [10,11].

#### 3.1.3. Wound Drainage and Fistula Formation

Purulent drainage from the wound site or the formation of a fistulous tract strongly indicates that esophageal contents are leaking into surrounding tissues. This is a critical warning sign of deep infection and may be associated with foul-smelling discharge if anaerobic organisms are present [9,12,14].

#### 3.1.4. Subcutaneous Emphysema

The detection of palpable crepitus in the neck indicates air escaping from the esophagus into the subcutaneous tissues, a hallmark of esophageal perforation. This finding may also be associated with radiographic evidence of air tracking along fascial planes [13,15].

#### 3.1.5. Systemic Inflammatory Response

Fever, tachycardia, hypotension, and an elevated white blood cell count (leukocytosis) are common findings. If the infection is not contained, these systemic signs may quickly progress to sepsis or septic shock. Hyperthermia or hypothermia, altered mental status, and elevated serum lactate levels may indicate a more severe systemic response requiring immediate intervention [11,12,16].

#### 3.1.6. Respiratory Compromise

In cases where esophageal contents aspirate the airway, patients may develop respiratory distress, aspiration pneumonia, or even acute respiratory distress syndrome (ARDS), further complicating the clinical picture. Stridor, wheezing, or decreased breath sounds may indicate airway involvement or compression [10,11].

#### 3.1.7. Additional Indicators

Severe throat pain, excessive salivation, retropharyngeal swelling, dysphonia, and foreign body sensation may also be present. Airway compression due to abscess formation or extensive swelling can lead to stridor and urgent respiratory compromise, necessitating airway management [9,13].

### 3.2. Late-Onset Symptoms (Days to Weeks Postoperatively)

**Figure 2:**
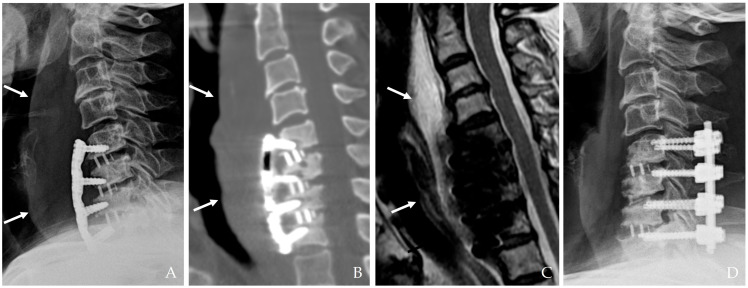
The patient underwent C5-6-7-T1 anterior cervical discectomy and fusion. At postoperative 6 days, postoperative infection occurred with retropharyngeal abscess and mediastinal extension (white arrows) (**A**–**C**). Therefore, the patient underwent evacuation of retropharyngeal abscess, irrigation/debridement, removal of plate/screws, and C5-6-7-T1 posterior fixation and achieved a cure for the infection (**D**).

If the initial esophageal injury is not promptly identified and managed, the infection may evolve into a more indolent yet destructive process over days or weeks (Figure 2) [10,11,12,13,14]. Late-onset symptoms include the following.

#### 3.2.1. Persistent Dysphagia and Recurrent Aspiration

Chronic difficulties with swallowing are indicative of ongoing esophageal dysfunction, possibly due to scarring, chronic inflammation, or a persistent fistula. Repeated aspirations can result in recurrent pneumonia or chronic lung disease if left untreated [11,12].

#### 3.2.2. Vocal and Laryngeal Changes

Hoarseness may develop because of recurrent laryngeal nerve involvement, either from direct inflammatory compression or secondary to abscess formation near the nerve. Progressive dysphonia or vocal cord paralysis may be evident in severe cases [10,15].

#### 3.2.3. Chronic Fistula Formation

A persistent fistulous tract can develop, leading to continuous leakage of esophageal contents into the neck and mediastinal spaces and perpetuating the infectious process. Fistulae are often associated with a non-healing wound, recurrent discharge, and chronic inflammation [13,14].

#### 3.2.4. Deep Neck Abscess and Mediastinitis

The infection may extend into deeper cervical tissues, forming abscesses that can coalesce and spread into the mediastinum. This is a life-threatening complication characterized by diffuse inflammation, tissue destruction, and multi-organ involvement (Figure 2) [10,12,14]. The time from surgery to abscess formation ranges from several days to weeks, and symptoms may include persistent fever, chest pain, and respiratory compromise.

#### 3.2.5. Systemic Sepsis

As the infection disseminates, patients may develop signs of severe sepsis or septic shock, including hypotension, altered mental status, and multi-organ dysfunction. Advanced diagnostic modalities are often required to identify the source of sepsis when symptoms are atypical or when multiple sites are involved [11,12,16].

#### 3.2.6. Hardware Compromise and Spinal Instability

In cases involving spinal instrumentation, prolonged infection can lead to loosening or extrusion of hardware, compromising the structural stability of the cervical spine. This complication may present with mechanical neck pain, deformity, or progressive neurological deficits [13,15].

#### 3.2.7. Nutritional Deficits and Dehydration

Chronic dysphagia often results in significant nutritional deficits, dehydration, weight loss, and immunosuppression, further impairing the patient’s overall recovery and increasing susceptibility to secondary infections [9,12].

## 4. Diagnosis

Early detection of esophageal injury and the ensuing postoperative infection is crucial for timely and effective intervention. A systematic diagnostic approach integrates clinical evaluation, advanced imaging techniques, and comprehensive laboratory testing [13,15,16,17,18,19,20,21,22,23,24]. Diagnostic strategies should be tailored to individual patients based on clinical presentation, risk factors, and suspected complications. The integration of multimodal diagnostic tools provides the highest sensitivity and specificity for identifying infection and guiding subsequent treatment.

### 4.1. Imaging Studies

#### 4.1.1. Computed Tomography (CT) Scanning

CT imaging with intravenous and oral contrast offers high-resolution images invaluable in detecting soft tissue infections, abscesses, mediastinitis, and retropharyngeal fluid collections [17,21,23]. CT can also reveal subtle signs such as extraluminal air, fluid accumulations, and hardware-related changes. This modality is essential for planning surgical interventions and assessing the full extent of the infection [13,15]. Recent advancements in CT technology, including dual-energy CT and iterative reconstruction techniques, have improved diagnostic accuracy and reduced radiation exposure, particularly important for patients requiring repeated imaging [16].

#### 4.1.2. Magnetic Resonance Imaging (MRI)

**Figure 3:**
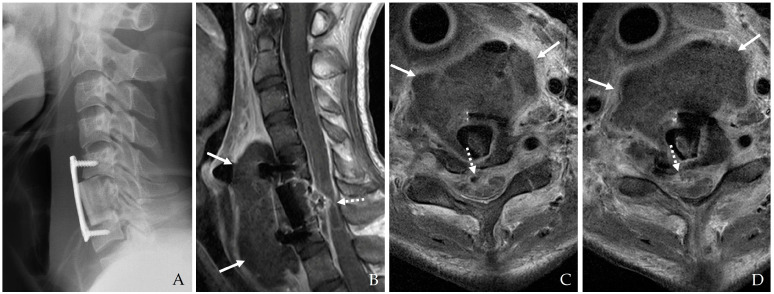
The patient underwent C5-6-7 anterior cervical discectomy and fusion (**A**). At postoperative 2 months, postoperative infection occurred, and magnetic resonance images showed retropharyngeal (white arrows) and epidural (dotted white arrows) (**B**–**D**).

MRI with contrast enhancement is highly effective in differentiating postoperative changes from active infectious processes, particularly when complications such as epidural abscess, osteomyelitis, or spinal cord compression are suspected [17,21,24]. MRI’s high soft tissue contrast resolution is indispensable for evaluating deep-seated infections (Figure 3). Diffusion-weighted imaging (DWI) and dynamic contrast-enhanced MRI are increasingly employed to improve sensitivity in detecting infection-related changes [15]. Furthermore, advanced imaging techniques, such as 3D reconstruction and volumetric assessment, can enhance the detection of subtle pathological changes and guide surgical planning [16] (Figure 3).

#### 4.1.3. Contrast Esophagography

**Figure 4:**
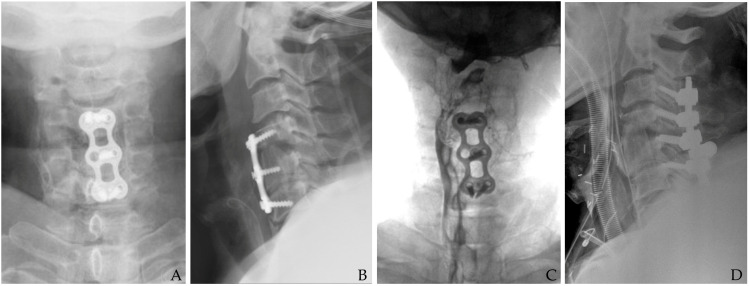
The patient underwent C5-6-7 anterior cervical corpectomy and fusion (**A**,**B**). At postoperative 3 days, postoperative infection occurred, and contrast esophagography showed esophageal rupture with dye leakage (**C**). Therefore, the patient underwent irrigation/debridement, removal of plate and screws, primary repair of esophageal rupture, and C3-4-5-6-7 posterior fixation (**D**). However, the patient died due to sepsis.

Contrast esophagography remains a cornerstone in diagnosing esophageal perforations and fistulas. The procedure involves using either barium or Gastrografin contrast agents to visualize the integrity of the esophageal lumen [18,22,23]. Although Gastrografin is generally preferred due to its lower risk of mediastinitis if extravasation occurs, barium is utilized in select cases because of its superior sensitivity [19,23]. This procedure is beneficial for detecting small leaks and determining the extent of perforation (Figure 4). Dynamic swallow studies and functional assessments are increasingly incorporated to provide a comprehensive evaluation of esophageal integrity and function [21].

#### 4.1.4. Flexible Esophagoscopy

**Figure 5:**
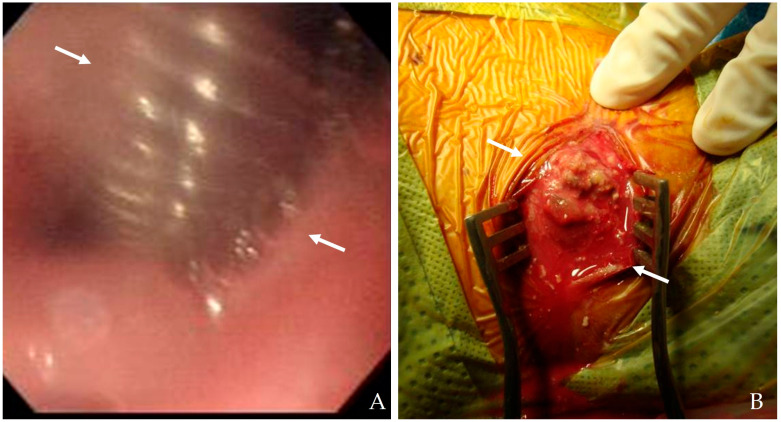
The patient underwent C5-6-7 anterior cervical discectomy and fusion (**A**). At postoperative 2 months, postoperative infection occurred, and flexible esophagoscopy showed esophageal rupture (white arrows) (**A**). Therefore, the patient underwent evacuation of retropharyngeal abscess (white arrows), irrigation/debridement, and primary repair of esophageal rupture (**B**).

Direct visualization through flexible esophagoscopy allows for identifying the exact location and size of mucosal tears, inflammatory changes, and esophageal defects [20,22]. Because this procedure avoids using contrast agents, it is particularly safe in patients with suspected perforations. Endoscopic evaluation facilitates therapeutic interventions in certain cases, such as stent placement (Figure 5). Innovations in endoscopic imaging, including high-definition endoscopy and narrow-band imaging, have enhanced diagnostic accuracy and enabled targeted therapeutic interventions [24].

#### 4.1.5. Supplementary Imaging Modalities

Additional imaging studies, such as fluoroscopic swallow studies, are sometimes used to assess functional swallowing impairment and guide rehabilitative efforts [15,21]. Ultrasound may be used for guiding percutaneous drainage of localized collections, and PET-CT can help identify low-grade or chronic infections in cases where conventional imaging is inconclusive [13,21]. Newer imaging modalities such as diffusion tensor imaging (DTI) and positron emission tomography–magnetic resonance imaging (PET-MRI) are being explored for their potential to provide enhanced diagnostic accuracy, particularly in differentiating infectious from inflammatory or neoplastic processes [16].

### 4.2. Laboratory Inflammatory Markers and Biomarkers

#### 4.2.1. C-Reactive Protein (CRP) and Erythrocyte Sedimentation Rate (ESR)

Elevated CRP and ESR levels are almost universally present in postoperative infections [17,23]. These markers help quantify the inflammatory response and monitor treatment efficacy over time. Procalcitonin is increasingly used to differentiate bacterial infections from other inflammatory conditions, especially in critically ill patients [18,21]. Serial measurements of these markers can help identify trends indicative of persistent infection or successful resolution [23].

#### 4.2.2. Complete Blood Count (CBC)

A CBC can provide critical information regarding the patient’s immunological status. An elevated white blood cell count, particularly with a neutrophilic predominance, is a classic sign of acute infection [19,23]. Conversely, lymphopenia or neutropenia may indicate severe systemic infection or immunosuppression [20,21]. Advanced techniques such as flow cytometry and immunophenotyping may provide additional insights into the immune response and guide targeted therapies [15].

#### 4.2.3. Microbiological Studies

Blood cultures, wound cultures, and Gram stains are essential for identifying causative pathogens [17,22]. These tests often reveal a polymicrobial infection, commonly involving Gram-positive cocci, Gram-negative bacilli, and anaerobic bacteria. Tailoring antibiotic therapy based on culture and sensitivity results is critical for effective management [18,23]. Novel diagnostic methods such as polymerase chain reaction (PCR), next-generation sequencing (NGS), and multiplex pathogen panels are increasingly being utilized to enhance pathogen detection and guide antibiotic stewardship [16].

### 4.3. Diagnostic Criteria for Postoperative Infection

The diagnosis of postoperative infection is based on a combination of clinical findings (persistent dysphagia, fever, neck pain, wound drainage, subcutaneous emphysema, and respiratory distress), supportive imaging evidence (contrast extravasation, fluid collections, abscess formation, and mediastinal involvement), and laboratory abnormalities (elevated inflammatory markers and positive cultures) [17,18,23]. A high index of clinical suspicion, particularly in patients with known risk factors, is essential for early diagnosis.

### 4.4. Additional Diagnostic Considerations

For patients with chronic or recurrent infections, advanced diagnostic modalities such as PET-CT scanning can be invaluable in detecting residual infection, osteomyelitis, or low-grade abscesses that may not be apparent on standard imaging [15,21]. Fluoroscopic swallow studies can assess the esophagus’s functional integrity post-repair, guiding further therapeutic and rehabilitative measures. Integration of all these diagnostic tools ensures a comprehensive assessment of the patient’s condition and informs subsequent treatment strategies [15,16,21].

## 5. Treatment Strategies

**Figure 6:**
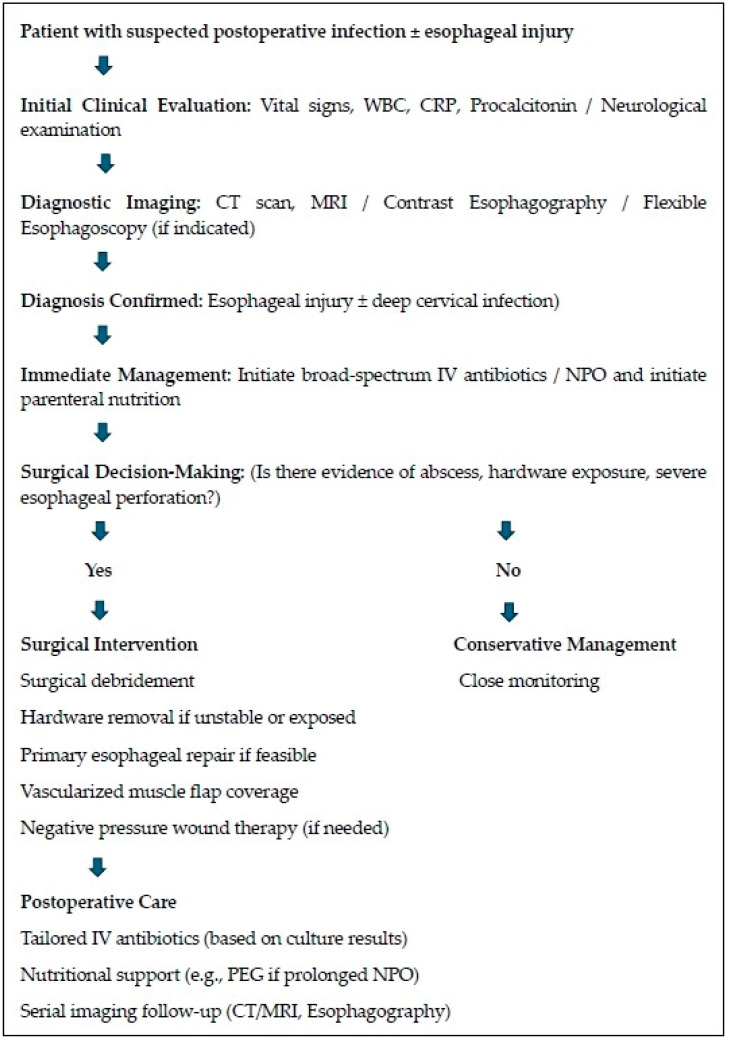
Algorithm for treatment strategy for postoperative infection with esophageal injury after anterior cervical spine surgery.

Management of postoperative infection in the context of esophageal injury requires a highly coordinated, multimodal approach that balances aggressive medical management with advanced surgical interventions [13,24,25,26]. Treatment strategies must be tailored to the patient’s specific clinical presentation, comorbidities, and the extent of the infection (Figure 6). Moreover, close coordination between surgical, medical, and rehabilitative teams is essential for optimizing outcomes and preventing recurrent infections.

### 5.1. Initial Management, Medical Stabilization, and Supportive Care

#### 5.1.1. Nutritional Support and Nothing by Mouth (NPO) Status

Patients are immediately placed on NPO status to prevent further surgical site contamination. Nutritional support is initiated using enteral feeding methods such as nasogastric (NG) or percutaneous endoscopic gastrostomy (PEG) tubes. When enteral feeding is not feasible—due to severe gastrointestinal dysfunction or intolerance, total parenteral nutrition meets the patient’s metabolic demands while supporting immune function and tissue healing [13,14,26]. In addition to providing nutritional support, maintaining an appropriate caloric intake is essential for promoting tissue healing, enhancing immune response, and preventing complications associated with malnutrition and muscle wasting.

#### 5.1.2. Broad-Spectrum Antibiotic Therapy

Early administration of empiric, broad-spectrum intravenous antibiotics is critical to control the infection. Common regimens include piperacillin-tazobactam or meropenem, often supplemented with vancomycin when methicillin-resistant Staphylococcus aureus is a concern. The choice of antibiotics is subsequently refined based on culture and sensitivity results, ensuring targeted therapy that minimizes the risk of antibiotic resistance and systemic toxicity [14,24,25]. Prolonged antibiotic therapy, both intravenous and oral, may be necessary in cases involving deep-seated infections, particularly those with hardware involvement.

#### 5.1.3. Hemodynamic Stabilization

Aggressive intravenous fluid resuscitation is initiated to stabilize the patient’s hemodynamics. In cases of septic shock, vasopressor support is provided to maintain adequate perfusion and prevent end-organ damage. Continuous monitoring in an intensive care setting is often required during this critical period [13,14]. Advanced hemodynamic monitoring techniques, including arterial pressure waveform analysis and echocardiography, may be employed to guide fluid therapy and optimize cardiac output.

#### 5.1.4. Image-Guided Drainage

When imaging studies reveal localized deep neck abscesses or mediastinal fluid collections, percutaneous drainage is performed under CT or ultrasound guidance. This minimally invasive approach helps reduce the infectious burden and serves as an adjunct to systemic antibiotic therapy [15,24,27]. Repeat imaging and drainage may be necessary when the initial intervention is incomplete or when there is evidence of ongoing infection.

### 5.2. Surgical Management

Surgical intervention is essential for definitive treatment, particularly in patients with significant esophageal injury and deep-seated infections. Surgical management is tailored based on the severity of the injury, extent of infection, and stability of the cervical spine [13,14,24,25,26,28,29].

#### 5.2.1. Esophageal Injury-Related Surgical Interventions

(1) Primary Repair with Vascularized Flap Reinforcement

In cases where an esophageal perforation is identified intraoperatively, immediate primary repair is performed. This involves direct closure of the defect using absorbable sutures. Reinforcement with a vascularized muscle flap is recommended to ensure a watertight repair and enhance tissue healing. The sternocleidomastoid (SCM) muscle flap is frequently used due to its proximity and robust blood supply [14,26]. In some cases, the longus colli or pectoralis major muscle flaps may be utilized, especially when more significant defects or extensive tissue loss are encountered [13,14].

(2) Aggressive Debridement and Diversion Techniques

Aggressive debridement of necrotic and infected tissues is mandatory for patients presenting with delayed diagnosis and established infection. Surgical debridement should extend beyond visibly affected areas to include all potentially compromised tissue [24,25]. Diversion procedures, such as inserting a feeding tube or NG tube for enteric diversion, are critical to minimize further contamination and allow the surgical repair to heal effectively [13].

(3) Complex Layered Closure

A complex, multilayered closure is required in scenarios involving large perforations or cases complicated by mediastinitis and hardware involvement. This approach involves the sequential closure of different tissue layers and is often reinforced with one or more vascularized muscle flaps [26]. An omental flap may be considered in extensive defects, as it provides a rich vascular network and immunologic benefits that can aid in infection control [14].

(4) Esophageal Diversion and Delayed Reconstruction

In severe cases where primary repair is not feasible, esophageal diversion (esophagostomy) may be performed. This temporizing measure diverts esophageal contents away from the injured area and is typically followed by delayed reconstruction once infection control has been achieved [13,14].

#### 5.2.2. Management of Superficial and Deep Infections and Instrumentation-Related Issues

The management of superficial and deep infections, particularly those involving spinal instrumentation, presents unique challenges that require a meticulous and multimodal approach. The use of advanced technologies and innovative therapeutic strategies has enhanced the ability to manage these complex infections effectively [13,14,15,24,25,26,27,28,29,30].

(1) Superficial Infections

Superficial infections may be managed conservatively with local wound care, including meticulous debridement, frequent dressing changes, and targeted antibiotic therapy. Negative pressure wound therapy (NPWT) is often used to enhance wound healing by reducing edema, improving perfusion, and promoting granulation tissue formation [15]. NPWT can also facilitate the removal of exudate, decrease bacterial load, and promote angiogenesis. Combining NPWT with adjunctive treatments such as antimicrobial dressings or silver-impregnated dressings can further optimize wound healing, particularly in patients with compromised immune status or poor nutritional intake.

(2) Deep Infections Without Instrumentation

In cases where deep infection is present without involvement of spinal hardware, extensive surgical debridement, irrigation, and drainage are necessary. Prolonged postoperative antibiotic therapy is critical to eradicate the infection and prevent recurrence [24,27]. Additionally, ensuring the complete removal of necrotic tissue and optimizing local perfusion are essential for preventing re-infection. Utilizing intraoperative culture techniques can guide antibiotic selection, enhancing targeted antimicrobial therapy.

(3) Deep Infections Involving Instrumentation

When the infection involves spinal hardware, the initial surgical approach may involve debridement while preserving the implants, provided the fixation remains stable. However, persistent or worsening infection frequently necessitates the removal of the hardware. In such cases, staged reconstruction may involve anterior or posterior spinal fusion using autologous bone grafts, allografts, or titanium cages [16,31]. This staged approach allows for the resolution of the infection before attempting definitive spinal stabilization. Newer technologies such as antibiotic-coated implants and biofilm-resistant materials are currently under investigation to improve outcomes in hardware-associated infections.

(4) Epidural Abscess and Osteomyelitis

**Figure 7:**
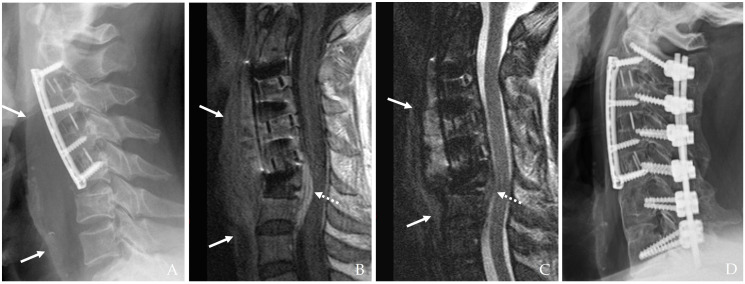
The patient underwent C2-3-4-5 anterior cervical discectomy and fusion (**A**). At postoperative 2 months, postoperative infection occurred with retropharyngeal abscess (white arrows) and epidural abscess (dotted white arrows) (**B**,**C**). Therefore, the patient underwent evacuation of retropharyngeal abscess, irrigation/debridement, and revisional C5-6-7 anterior cervical discectomy and fusion and C2-3-4-5-6-7 posterior fixation (**D**) and achieved a cure for the infection.

The presence of an epidural abscess or osteomyelitis requires urgent surgical intervention. In these situations, decompressive laminectomy or corpectomy with subsequent fusion may be required to relieve pressure on the spinal cord and eradicate the infection [15,27]. Aggressive debridement is paramount to prevent permanent neurological deficits and further spread of infection (Figure 7). Long-term antibiotic therapy following surgical intervention is often necessary to ensure complete eradication of the pathogen, particularly in patients with osteomyelitis.

(5) Additional Adjuncts and Advanced Techniques

Recent advancements in surgical technology have introduced adjunctive therapies that significantly improve outcomes. For example, NPWT and hyperbaric oxygen therapy (HBOT) may further aid in infection control and tissue healing by enhancing tissue oxygenation, accelerating wound healing, and exerting bactericidal effects against anaerobic pathogens [15,27]. Other novel techniques include photodynamic therapy and ultrasound-assisted debridement, which have shown potential in enhancing bacterial clearance and promoting tissue regeneration. Intraoperative neuromonitoring and image-guided navigation systems improve the debridement and hardware removal precision, reducing the risk of collateral damage to critical structures.

(6) Antibiotic-Loaded Cement Beads

**Figure 8:**
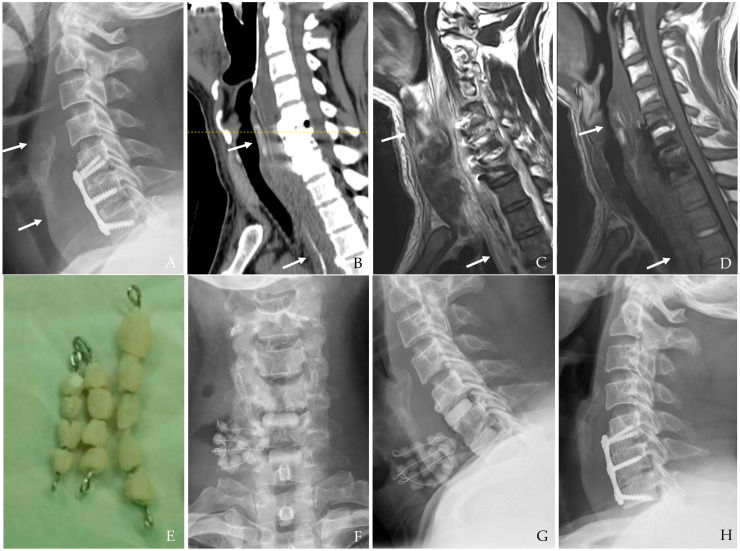
The patient underwent C5-6-7 anterior cervical discectomy and fusion (**A**). At postoperative 3 days, postoperative infection occurred with retropharyngeal abscess and mediastinal extension (white arrows) (**B**–**D**). Therefore, the patient underwent the removal of the anterior plate and screws, evacuation of retropharyngeal abscess, irrigation/debridement, and antibiotic-loaded cement beads (**E**–**G**). The patient underwent C5-6-7 revisional anterior cervical discectomy and fusion 2 weeks after reoperation and achieved a cure for the infection (**H**).

In the case of deep surgical site infections, particularly those involving hardware-related infections, antibiotic-loaded cement beads can be an effective adjunct to systemic antibiotic therapy [16,31,32,33,34]. These beads provide localized, high-concentration antibiotic delivery to the infected site while minimizing systemic toxicity. Their use is particularly beneficial in (a) persistent deep infections resistant to standard antibiotic therapy, (b) cases where hardware preservation is desired, as the beads can help suppress infection without immediate implant removal, and (c) post-debridement infection control, helping prevent bacterial recolonization in the surgical cavity. Common antibiotics used in cement beads include vancomycin, gentamycin, and tobramycin, tailored to the suspected or confirmed pathogens. The beads may be placed temporarily and later removed once infection control is achieved or may serve as a bridge before definitive surgical reconstruction (Figure 8). Newer biodegradable antibiotic carriers are under investigation to improve drug delivery and minimize the need for secondary procedures for removal.

#### 5.2.3. Enhanced Focus on Surgical Techniques and Multidisciplinary Coordination

The complexity of managing esophageal injuries with concomitant postoperative infection necessitates close collaboration among various surgical specialties. Multidisciplinary teams comprising spine surgeons, otolaryngologists, gastroenterologists, infectious disease specialists, radiologists, intensivists, and rehabilitation experts are essential for achieving optimal outcomes. Coordination of care ensures that infection control, esophageal repair, and spinal stabilization are achieved in a manner that minimizes overall morbidity and improves functional recovery [15,24,25]. Effective communication among the multidisciplinary team is essential for developing a comprehensive treatment plan that addresses the multifaceted nature of postoperative infection and esophageal injury. Regular interdisciplinary meetings, coordinated treatment protocols, and shared decision-making enhance the ability to deliver tailored, patient-specific care.

Surgeons often follow staged protocols where initial procedures focus on infection control and esophageal repair, with subsequent reconstructive surgeries performed once the infectious process has been adequately contained. This approach improves outcomes and preserves the structural and functional integrity of the cervical spine and esophagus. Additionally, implementing perioperative optimization strategies, such as nutritional support, antimicrobial stewardship, and enhanced recovery after surgery (ERAS) protocols, contributes to improved patient outcomes and reduced complication rates. Advanced surgical techniques, including minimally invasive approaches, robotic-assisted surgery, and enhanced intraoperative imaging, are increasingly being utilized to improve precision and reduce surgical morbidity. These innovations allow for better visualization of the surgical field, more accurate debridement, and improved placement of vascularized flaps for esophageal repair. Moreover, technological advancements in intraoperative neuromonitoring and navigation systems help reduce the risk of iatrogenic injury and enhance surgical accuracy.

Furthermore, long-term follow-up and rehabilitation are integral components of the multidisciplinary approach. Rehabilitative strategies may include speech and swallowing therapy, physical therapy, and nutritional counseling to optimize recovery and enhance the patient’s overall quality of life. Multidisciplinary clinics and coordinated follow-up care protocols can facilitate early detection of complications and prompt intervention when necessary. The integration of advanced surgical techniques, multidisciplinary coordination, and comprehensive postoperative care is essential for achieving successful outcomes in patients with complex postoperative infections involving esophageal injury. Continued research and refinement of these approaches are necessary to further improve patient outcomes and establish standardized treatment protocols.

## 6. Postoperative Care and Follow-Up

Effective postoperative care and follow-up are essential components in minimizing complications and improving patient outcomes following anterior cervical spine surgery. This section has been expanded to incorporate recent publications from the last five years (2020 to 2025), providing updated evidence-based recommendations and highlighting advancements in antibiotic therapy, imaging modalities, rehabilitation strategies, and long-term follow-up [13,14,15,16,21,24,26,27,28,29,30,34,35,36]. The implementation of a structured, multidisciplinary approach involving various medical professionals has been shown to enhance recovery rates, reduce recurrence of infections, and optimize long-term functional outcomes. This comprehensive management strategy is particularly relevant for patients with esophageal injuries or other complex postoperative complications.

### 6.1. Prolonged Antibiotic Therapy and Medical Management

#### 6.1.1. Extended Intravenous Antibiotic Regimen

Patients typically receive intravenous antibiotics for 6 to 8 weeks postoperatively to ensure complete eradication of the infection [35,36]. Following this intensive period, many patients are transitioned to oral suppressive antibiotic therapy to reduce the risk of recurrence, especially subjects with residual infection or compromised immunity. Recent studies have also emphasized the use of antibiotic-impregnated beads and microcomposite implants for controlled antibiotic delivery, enhancing infection control and promoting tissue healing [16,35]. Additionally, novel delivery systems such as antibiotic-eluting scaffolds and local injection therapies are being investigated for their efficacy in preventing recurrent infections and reducing systemic antibiotic exposure [34].

#### 6.1.2. Nutritional Support

Due to the frequent occurrence of prolonged dysphagia and malnutrition, sustained nutritional support is essential. Enteral feeding via NG or PEG tubes is maintained until the patient demonstrates adequate swallowing function and can resume regular oral intake. Nutritional support promotes wound healing and bolsters the patient’s immune response during recovery [13,26]. Furthermore, enhanced nutritional strategies involving immunomodulatory nutrition (e.g., arginine, omega-3 fatty acids, and glutamine) have been suggested to improve healing rates and reduce infection-related complications [14,16].

### 6.2. Regular Imaging and Laboratory Monitoring

#### 6.2.1. Serial Imaging Studies

Follow-up imaging using CT, MRI, or fluoroscopic swallow studies is routinely performed to monitor the resolution of the infection and assess the integrity of esophageal repair and spinal constructs [21,24]. Enhanced imaging modalities such as PET/CT have demonstrated improved diagnostic accuracy in detecting musculoskeletal infections and assessing the extent of inflammatory processes [16,21]. Novel imaging techniques, including diffusion-weighted imaging (DWI) and dynamic contrast-enhanced MRI, are being explored for their potential to provide even greater specificity and sensitivity in infection detection [34].

#### 6.2.2. Laboratory Surveillance

Monitoring inflammatory markers (CRP, ESR, and procalcitonin) and CBC is essential to track the patient’s response to therapy. Declining inflammatory markers are an encouraging sign of infection resolution; however, persistent or rising levels may indicate the need for further investigation or therapeutic adjustments [13,24,35]. Additionally, recent research suggests the inclusion of novel biomarkers such as interleukin-6 (IL-6) and proadrenomedullin (PCT) for improved diagnostic accuracy and monitoring of treatment efficacy [16,21,34].

### 6.3. Rehabilitation and Functional Recovery

#### 6.3.1. Speech and Swallowing Therapy

Early initiation of speech and swallowing therapy is critical in patients undergoing esophageal repair. Therapists work closely with patients to re-establish standard swallowing mechanics, reduce aspiration risk, and improve overall quality of life [13,14]. Intensive rehabilitative efforts may include exercises to strengthen the oropharyngeal muscles and techniques to compensate for altered swallowing physiology. Recent guidelines emphasize the importance of personalized therapy protocols, incorporating both conventional and novel rehabilitation technologies, such as neuromuscular electrical stimulation (NMES) and virtual reality-based therapies [34].

#### 6.3.2. Physical Therapy and Cervical Rehabilitation

Concurrent with swallowing therapy, patients often require physical therapy to regain strength, improve range of motion, and stabilize the cervical spine. Tailored rehabilitation programs are designed with spine surgeons and physiatrists to protect surgical repairs and spinal fusions while promoting functional recovery [14,26]. The integration of functional outcome assessments, including validated scoring systems and objective performance metrics, is increasingly recommended to optimize recovery protocols [16,34].

### 6.4. Management of Late Complications and Long-Term Follow-Up

#### 6.4.1. Esophageal Stricture Management

Scar formation and chronic inflammation can lead to the development of esophageal strictures that impede normal swallowing. Endoscopic dilatation is the primary treatment for such strictures, and multiple sessions may be necessary to achieve a stable resolution [24,26]. More advanced interventions, such as stenting or surgical revision, may be considered in refractory cases. Newer technologies, including biodegradable stents and tissue engineering approaches, are currently under investigation to improve outcomes in difficult cases [16,34].

#### 6.4.2. Regular Multidisciplinary Follow-Up

Ongoing follow-up with a multidisciplinary team, including spine surgeons, gastroenterologists, infectious disease specialists, and rehabilitation experts, is crucial for monitoring long-term outcomes [13,21]. Regular clinical evaluations, imaging studies, and laboratory tests are integrated into the follow-up schedule to detect and promptly address emerging complications. Enhanced coordination between specialists and improved communication protocols are being developed to streamline follow-up care [35,36].

#### 6.4.3. Psychosocial Support and Nutritional Counseling

Due to the prolonged recovery process and potential impact on quality of life, patients often benefit from psychosocial support and nutritional counseling [13,26]. These services help address the emotional and dietary challenges associated with long-term dysphagia, chronic pain, and changes in body image following extensive surgical procedures. Additionally, recent studies advocate the inclusion of mental health screening and intervention as part of routine postoperative care to enhance overall recovery and patient satisfaction [34,35].

## 7. Prognosis, Complications, and Prevention

The prognosis for patients with postoperative infection complicated by esophageal injury is highly contingent upon the timeliness of diagnosis and aggressiveness of the treatment regimen. Early intervention is critical in halting the spread of infection and preventing irreversible damage to the esophagus and cervical spine (Table 1) [1,3,11,13,14,24,25,26]. Additionally, recent literature emphasizes the importance of long-term monitoring and preventive measures tailored to individual risk factors to optimize recovery and minimize complications [35,36].

### 7.1. Prognostic Factors

#### 7.1.1. Timeliness of Diagnosis and Treatment

Rapid recognition of esophageal injury and immediate medical and surgical management implementation significantly improve patient outcomes. Delays in diagnosis often lead to widespread infection, mediastinitis, and systemic sepsis, which are associated with a markedly poorer prognosis. Increasing evidence supports the use of advanced imaging modalities and biomarkers such as interleukin-6 (IL-6) and proadrenomedullin (PCT) to enhance early detection and monitoring of treatment efficacy [13,16,21,34].

#### 7.1.2. Extent of Infection and Tissue Damage

The degree of soft tissue involvement, the presence of deep abscesses, and the extent of vertebral destruction are key determinants of long-term outcomes. Patients with limited localized infections tend to have better prognoses than individuals with extensive mediastinal or epidural involvement. Additionally, the presence of necrotic tissue and compromised blood supply can significantly hinder the healing process, making early debridement and vascularized tissue reinforcement essential [14,24,26].

#### 7.1.3. Patient-Related Factors

Underlying comorbidities such as diabetes, obesity, and immunosuppression can adversely affect the healing process and increase the risk of recurrent infections. Adequate nutritional status and the patient’s overall physiological reserve are also critical for recovery. Recent studies have highlighted the importance of assessing patient frailty and immune status before initiating aggressive surgical interventions, with tailored perioperative care shown to improve outcomes in high-risk populations [35,36].

### 7.2. Potential Complications

#### 7.2.1. Chronic Dysphagia and Esophageal Strictures

Even after successful initial treatment, patients may experience persistent dysphagia due to chronic inflammation, scarring, and stricture formation. These complications often necessitate repeated endoscopic interventions and long-term dietary modifications. The incorporation of novel dilation techniques, including balloon-assisted and stent-assisted dilation, has demonstrated improved efficacy in managing refractory strictures [13,14].

#### 7.2.2. Recurrent Fistula Formation

In cases where esophageal repair is complicated by ongoing infection, recurrent fistulae may develop, leading to continuous leakage and necessitating further surgical revision. The use of tissue engineering approaches, including biologic scaffolds and stem cell-based therapies, is being explored as a means of enhancing tissue regeneration and preventing recurrent fistula formation [24,34].

#### 7.2.3. Hardware-Related Complications

Infection involving spinal instrumentation can lead to hardware loosening, extrusion, or even complete failure of the fixation system. Such complications compromise spinal stability and require additional surgical interventions, often involving staged reconstruction and fusion. Prophylactic measures, such as coating implants with antimicrobial agents or using antibiotic-loaded cement, are being actively investigated to reduce infection rates associated with hardware use [16,35].

#### 7.2.4. Systemic Sequelae

Patients who experience delayed or inadequate treatment may develop systemic sepsis, multi-organ dysfunction, and long-term neurological deficits. In particular, mediastinitis remains one of the most feared complications due to its high mortality rate. Improved critical care management, including early sepsis protocols and optimized antibiotic regimens, is essential for mitigating these risks [11,13,14].

### 7.3. Preventative Strategies

#### 7.3.1. Meticulous Surgical Technique

The cornerstone of prevention lies in the careful handling of tissues during surgery. Minimizing esophageal traction and using protective soft tissue interposition techniques, such as SCM muscle flaps or allografts, are critical for reducing the risk of injury. Additionally, recent advancements in minimally invasive techniques and robotic-assisted surgeries are being explored to reduce iatrogenic injury and improve surgical precision [1,3,24].

#### 7.3.2. Perioperative Antibiotic Prophylaxis

Strict adherence to perioperative antibiotic protocols significantly reduces the incidence of postoperative infections. In high-risk patients, extended prophylactic regimens may be warranted [35,36]. Novel strategies involving localized antibiotic delivery systems, such as impregnated beads or antibiotic-coated implants, are currently under investigation to enhance infection control without increasing systemic toxicity [16,35].

#### 7.3.3. Early Postoperative Assessment

Vigilant monitoring for signs of esophageal compromise, such as dysphagia, subcutaneous emphysema, or unexpected wound drainage, enables early intervention. Routine postoperative imaging and clinical evaluations are essential for identifying complications before they become life-threatening. Implementing standardized follow-up protocols and integrating biomarkers into routine assessments may further enhance diagnostic accuracy and improve patient outcomes [13,21,34].

## 8. Conclusions

Postoperative infection following anterior cervical spine surgery, particularly when complicated by concomitant esophageal injury, poses a significant clinical challenge that requires prompt recognition, accurate diagnosis, and comprehensive management. Our review emphasizes the necessity of adopting a multidisciplinary approach that integrates advanced diagnostic techniques, surgical debridement, vascularized flap reinforcement, negative pressure wound therapy, and antibiotic-loaded cement beads. The appropriate use of imaging modalities such as computed tomography, magnetic resonance imaging, contrast esophagography, and flexible esophagoscopy plays a crucial role in the timely diagnosis and treatment planning of these complex cases.

Meticulous postoperative care is essential for minimizing recurrence and optimizing clinical outcomes. This includes prolonged antibiotic therapy, nutritional support, regular imaging follow-up, and collaboration among spine surgeons, infectious disease specialists, and rehabilitation teams. Moreover, recent innovations such as hyperbaric oxygen therapy and vascularized muscle flap reinforcement have demonstrated promise in enhancing tissue healing and improving infection control.

Future research should focus on conducting well-designed meta-analyses to provide statistically robust evidence for refining treatment protocols. Additionally, standardized guidelines for managing postoperative infections associated with anterior cervical spine surgery, particularly those involving esophageal injury, are warranted. Continued advancements in diagnostic tools, surgical techniques, and postoperative care are essential for minimizing complications and improving patient outcomes.

Achieving optimal clinical outcomes requires a proactive approach that integrates evidence-based practices, technological innovations, and collaborative efforts among healthcare professionals. This comprehensive review is a guideline for enhancing treatment outcomes and preventing recurrence in patients experiencing postoperative infections following anterior cervical spine surgery.

## Figures and Tables

**Table 1:** Prognosis, Complications, and Prevention.

Study	Year	Study Design	Key Findings	Reference
Alhashash et al.	2023	Retrospective database analysis	Identified risk factors including diabetes, immunosuppression, and implant use.	[1]
Spatenkova et al.	2023	Single-center cohort study	Emphasized the importance of multimodal preventive strategies.	[3]
Bivona et al.	2023	Case series	Described clinical features of delayed retropharyngeal and parapharyngeal abscesses.	[11]
Yuan et al.	2017	Retrospective study	Highlighted early intervention for esophageal fistula prevention.	[25]
Sun et al.	2012	Cohort study	Provided preventive strategies for esophageal fistula.	[13]
Kang et al.	2017	Retrospective study	Discussed management of esophageal and pharyngeal perforation.	[26]
Orlando et al.	2003	Case series	Provided guidelines for cervical esophagus management.	[24]
Navarro et al.	2005	Case report	Described the use of SCM muscle flap for esophageal repair.	[14]

## Data Availability

Not applicable.

## Abbreviations

The following abbreviations are used in this manuscript:

CT	Computed Tomography
MRI	Magnetic Resonance Imaging
CRP	C-Reactive Protein
ESR	Erythrocyte Sedimentation Rate
CBC	Complete Blood Count
SCM	Sternocleidomastoid
NPO	Nothing By Mouth
NG	Nasogatric
PEG	Percutaneous Endoscopy Gastrostomy
NPWT	Negative Pressure Wound Therapy
HBOT	Hyperbaric Oxygen Therapy
